# The Effects of Temperature on the Stability of a Neuronal Oscillator

**DOI:** 10.1371/journal.pcbi.1002857

**Published:** 2013-01-10

**Authors:** Anatoly Rinberg, Adam L. Taylor, Eve Marder

**Affiliations:** Volen Center and Biology Department, Brandeis University, Waltham, Massachusetts, United States of America; Research Center Jülich, Germany

## Abstract

The crab *Cancer borealis* undergoes large daily fluctuations in environmental temperature (8–24°C) and must maintain appropriate neural function in the face of this perturbation. In the pyloric circuit of the crab stomatogastric ganglion, we pharmacologically isolated the pacemaker kernel (the AB and PD neurons) and characterized its behavior in response to temperature ramps from 7°C to 31°C. For moderate temperatures, the pacemaker displayed a frequency-temperature curve statistically indistinguishable from that of the intact circuit, and like the intact circuit maintained a constant duty cycle. At high temperatures (above 23°C), a variety of different behaviors were seen: in some preparations the pacemaker increased in frequency, in some it slowed, and in many preparations the pacemaker stopped oscillating (“crashed”). Furthermore, these crashes seemed to fall into two qualitatively different classes. Additionally, the animal-to-animal variability in frequency increased at high temperatures. We used a series of Morris-Lecar mathematical models to gain insight into these phenomena. The biophysical components of the final model have temperature sensitivities similar to those found in nature, and can crash via two qualitatively different mechanisms that resemble those observed experimentally. The crash type is determined by the precise parameters of the model at the reference temperature, 11°C, which could explain why some preparations seem to crash in one way and some in another. Furthermore, even models with very similar behavior at the reference temperature diverge greatly at high temperatures, resembling the experimental observations.

## Author Summary

The nervous systems of cold-blooded animals must maintain essential function despite fluctuations in environmental temperature. We studied the pyloric rhythm of the crab, *Cancer borealis*. The pyloric rhythm is important for the animal's feeding behavior, and previous work has shown that relative timing, or phase, of the neurons in the pyloric circuit is temperature invariant over a range of physiologically realistic temperatures (7 to 23°C), although the frequency of the rhythm increases. At higher temperatures, the rhythm often becomes disorganized or stops. In this paper we present experimental work on the isolated pacemaker of the pyloric rhythm together with a computational model that explores the loss of stability of the pacemaker as a function of temperature. Experimentally, we found that the isolated pacemaker responds to temperature similarly to the intact network, and maintains constant duty cycle over large temperature ranges. At high temperatures, about half of the pacemakers stop oscillating, reminiscent of certain mathematical bifurcations. We varied the temperature dependence and conductance densities of a simple model oscillator, and characterized its bifurcations as a function of temperature. We found that particular temperature-dependent relationships must be maintained to provide robust temperature performance of oscillators with variable underlying conductances.

## Introduction

Neuronal oscillators depend on the balanced interaction of many voltage-dependent currents to produce functional output. For example, the cardiac action potential is a result of the voltage- and time- dependent activation and inactivation of many different ion channels [1]. Likewise, thalamic neurons generate bursts that also depend on the properties of many currents [2]–[4].

Temperature is a global perturbation that influences the conductance, activation, and inactivation of all ion channels [5]. Because the temperature sensitivities of different ion channels are generally different, this variability presents a potential challenge to maintaining stable oscillatory function over an extended temperature range, as is necessary for neuronal oscillators found in cold-blooded animals.

A number of theoretical studies have shown that similar neuronal and network behaviors can be produced by widely different sets of conductances [6]–[9]. More specifically, very similar patterns of neuronal bursting can arise from different balances of inward and outward currents [7], [10]. Thus, even if temperature alters the relative balance of inward and outward currents in a neuronal oscillator, this divergence might not immediately lead to a loss of robust oscillation.

In the stomatogastric ganglion (STG) of the crab, *Cancer borealis*, the anterior burster (AB) neuron is strongly oscillatory, and is electrically coupled to the two pyloric dilator (PD) neurons. Together the AB and PD neurons comprise a three-neuron pacemaker kernel that drives the pyloric rhythm of the STG [11]. Previous work on the STG has shown that there is substantial variability in ionic currents across animals [12], [13]. Despite this variability, in a “permissive” temperature range (7°C to 23°C), the pyloric rhythm exhibits remarkably stable phase relationships among activity in different neurons [14]. However, at higher temperatures the rhythm often “crashes”, i.e. fails to oscillate [15]. Crashed preparations resume oscillations if returned to a permissive temperature.

In this paper we characterize the effects of temperature on the isolated pacemaker kernel of the pyloric rhythm, both over the permissive temperature range and at more extreme temperatures, as a way of probing the diversity of the underlying oscillatory mechanisms across individual animals. Additionally, by studying the effects of temperature on a simple oscillator model, we describe the generic features that enable neuronal oscillators to respond to temperature modifications in a reliable fashion.

## Results

**Figure 1:**
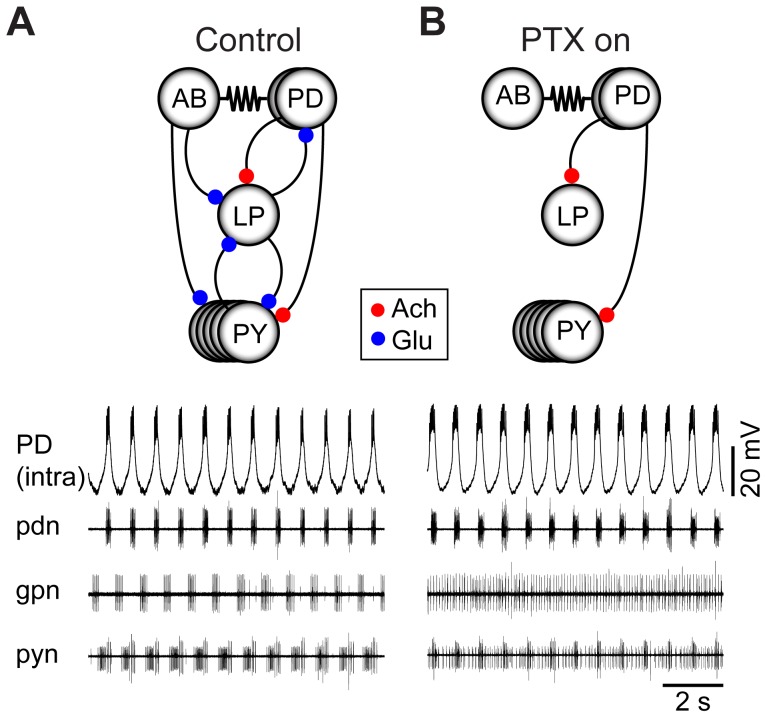
The pyloric network, intact and with pacemaker isolated. (A) At top, a schematic of the intact pyloric network. Dots represent inhibitory chemical synapses, resistor symbols indicates electrical synapses. Dot color represents the transmitter used by particular synapses. ACh = acetylcholine, Glu = glutamate. At bottom, intracellular recording of PD and simultaneous extracellular recordings from three nerves: *pdn*, *gpn*, and *pyn*, which reflect activity in PD, LP, and PY, respectively. Traces recorded at 11°C. (B) At top, a schematic of the pyloric network in presence of 10^−5^ M PTX, which blocks glutamatergic synapses in *C. borealis*. In this condition, the major synaptic input to the pacemaker from other pyloric neurons has been blocked. At bottom, the same preparation as in panel A, but after application of 10^−5^ M PTX. Deprived of pacemaker input, LP and PY fire tonically, but the pacemaker continues to oscillate.

The pyloric rhythm is a triphasic motor pattern in which the pacemaker kernel, consisting of the AB and two PD neurons, fires in alternating bursts with the lateral pyloric (LP) and pyloric (PY) neurons. The connectivity among the pyloric network neurons and the rhythm itself are shown in Figure 1A. Activity of the PD neurons can be seen in the intracellular recording of one of the PD neurons and on the extracellular recording from the pyloric dilator nerve (*pdn*; Figure 1A). Activity of the LP neuron is seen as the spikes recorded on the gastropyloric nerve (*gpn*; Figure 1A). The smaller amplitude spikes on the pyloric nerve (*pyn*) show activity from the PY neurons (Figure 1A). Note that while the LP and PY neurons are inhibited by both the glutamatergic AB and the cholinergic PD neurons [16], the only feedback to the pacemaker kernel comes from the glutamatergic LP neuron [17].

In the STG, picrotoxin (PTX) blocks the glutamatergic inhibitory synapses [16], [18], [19], thus allowing the pacemaker kernel to be isolated from other members of the pyloric circuit (Figure 1B). In the presence of 10^−5^ M PTX the pacemaker kernel maintains its activity, as seen in the intracellular PD recording and the extracellular *pdn* recording (Figure 1B). Loss of the AB inhibition usually causes the LP and PY neurons to fire tonically (the residual cholinergic inhibition from the PD neurons is often very weak). Because of the strong electrical coupling between the AB and PD neurons, PD neuron activity is a good monitor of the AB neuron's activity [11].

### Variability in pacemaker frequency at high temperature

**Figure 2:**
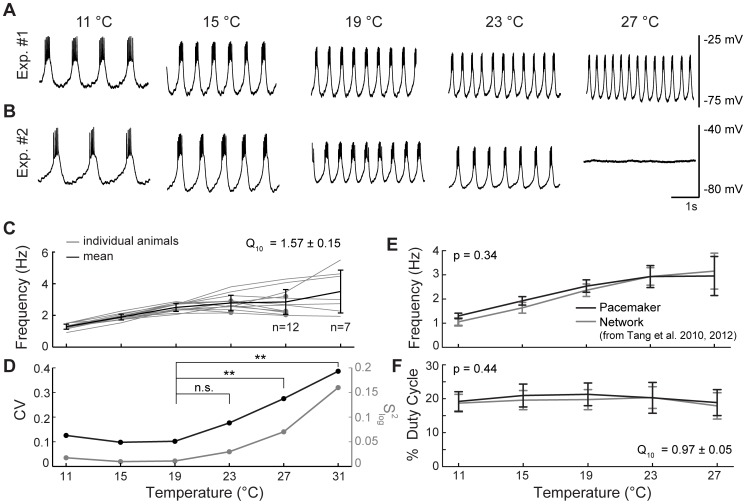
Effect of temperature on frequency and duty cycle of the isolated pyloric pacemaker. (A) Membrane potential of the PD neuron in one animal, at several temperatures. (B) Similar to A, but in a different individual. (C) PD burst frequency versus temperature for n = 14 individuals (gray), and averaged across individuals (black). Gray lines that end in a dot represent animals that crashed above that temperature. Error bars represent SD. (D) The coefficient of variation of the burst frequency (across individuals) at each temperature, and the variance of the log-transformed frequency (S^2^_log_, see Methods). Brackets denote a Levene *s* test to compare the log-transformed variance between two temperatures: n.s. = not significant, ** = p<0.01. (E) Pyloric frequency versus temperature, for the isolated pacemaker (black, subset of the data shown in panel C), and the intact network (gray, n = 15, as previously reported in [14], [15]). Data for 31°C is not shown because many preparations crashed or cycled erratically at this temperature. Error bars represent SD. (F) Duty cycle versus temperature, again in the isolated pacemaker (black, n = 12) and the whole network (gray, n = 15, as previously reported in [14], [15]). Again, data for 31°C is not shown. Error bars represent SD.

We studied the output of isolated pyloric pacemakers in a temperature range from 11 to 31°C (Figure 2). Generally, the frequency increased with temperature (Figure 2A). However, bursting was quite variable at extreme temperatures, and some individuals displayed a decrease in frequency as temperature was increased (Figure 2B). Furthermore, some preparations continued to cycle at 31°C, the highest temperature tested, whereas some crashed before this point (compare Figure 2A, 2B). Three isolated pacemaker kernels increased their frequency over the entire range from 11 to 31°C (as in Figure 2A). Seven isolated pacemakers crashed (as in Figure 2B). Finally, four isolated pacemakers showed frequency-temperature (F-T) curves that flattened out or sloped downward at high temperatures (as in Figure 2B, compare 19°C panel to 23°C panel).

Averaged across individuals, frequency increased with temperature (Figure 2C; *Q_10_* = 1.57±0.15, SE; See Methods). This increase was approximately linear from 11 to 19°C (slope = 0.152 Hz/°C; r^2^ = 0.87, 95% CI = [0.76, 0.93]). Variability of the frequency (across individuals) also increased with increasing temperature (Figure 2D; p<10^−4^, Levene *s* test on log-transformed frequency), with most of the change occurring above 19°C. From 11 to 19°C, variability of the frequency did not change significantly (p = 0.11, Levene *s* test on log-transformed frequency), but at higher temperatures the variability increased strongly (see Figure 2D for detailed comparisons).

Previously, it was shown that in the intact pyloric network, frequency increased with increasing acute temperature [14], [15]. Remarkably, we found that the isolated pacemaker had an F-T curve that was statistically indistinguishable from that of the intact network (Figure 2E, with data from [14], [15] shown for comparison; p = 0.34, two-way RM ANOVA).

### As in the intact network, isolated pacemaker duty cycle remains invariant to temperature

We measured the effect of temperature on the pacemaker duty cycle. For a bursting neuron, duty cycle is defined as the fraction of the period that the oscillator is bursting. In contrast to the effect of temperature on frequency, the isolated pacemaker kernel's duty cycle changed little as temperature was increased, exhibiting a *Q_10_* close to one (Figure 2F; *Q_10_* = 0.97±0.05; SE). In the previous work on the intact pyloric network, it was also found that the pacemaker duty cycle did not vary with temperature [14], [15]. As with frequency, we found that the isolated pacemaker had a duty-cycle- versus-temperature curve that was statistically indistinguishable from that of the intact network (Figure 2F, with data from [14], [15] shown for comparison; p = 0.44, two-way RM ANOVA). Of these preparations, some crashed at 31°C, but even these maintained constant duty cycle until they crashed. Together, these observations strongly suggest that the underlying mechanism for the pacemaker's duty-cycle invariance is intrinsic to the pacemaker itself and does not depend on network interactions.

### Crash characterization

**Figure 3:**
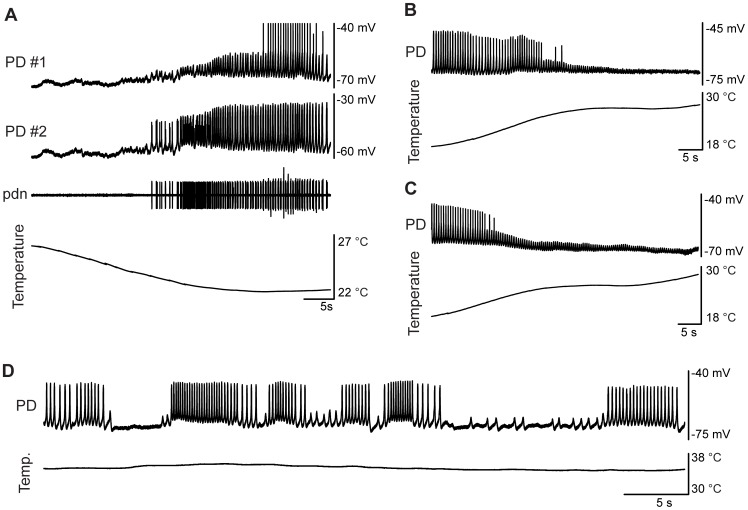
Examples of isolated pacemaker crashes at high temperatures. (A) Simultaneous intracellular recording from two PD neurons, and an extracellular recording from the *pdn*, as temperature is dropped from 27 to 23°C. In the top trace, spikes were cut off at −40 mV. (B, C) Two examples of similarly behaving oscillations: as temperature was increased, from around 18 to 30°C, amplitude dropped and frequency continued to increase, eventually terminating in small-amplitude oscillations. (D) PD recording at a steady 35°C shows multiple switching between oscillation and flat-line voltage seemingly through fold limit cycle bifurcations.

For each preparation, we attempted to push the system to its critical temperature, the temperature at which the behavior of the oscillations changed qualitatively, e.g. from robust oscillations to a fixed voltage. While seven isolated pacemakers crashed at or below 31°C, they showed a variety of behaviors as they transitioned in and out of robust bursting. Figure 3A shows a crash recovery in which the initially quiescent pacemaker transitioned to small-amplitude oscillations that then grew to larger amplitude as temperature was decreased. Figure 3B and C show a similar type of behavior (in the reverse direction) in which full-amplitude oscillations gradually decreased in amplitude as temperature was increased, while maintaining an approximately steady frequency. Qualitatively, this type of behavior is reminiscent of a supercritical Hopf bifurcation, in which amplitude transitions gradually from fixed voltage to full oscillations and at the transition point the oscillations are born at non-zero frequency [20].

In contrast, some preparations abruptly transitioned from full-amplitude oscillations to quiescence at high temperatures. In the example shown in Figure 3D, the rhythm spontaneously flipped between full-amplitude oscillations and quiescence at a fixed temperature. This kind of behavior is reminiscent of a fold limit cycle bifurcation, in which oscillations emerge at a non-zero amplitude [20]. Additionally, the sudden transitions at a fixed temperature suggest that the system is highly sensitive to small perturbations when it is close to a critical temperature.

### A model of the pyloric pacemaker

Broadly speaking, the experimental data show that as temperature is increased, the pacemaker duty cycle is largely constant while its frequency increases. Nevertheless, the individual preparations showed considerable diversity at high temperature, with some steadily increasing in frequency while others leveled off or even slowed (Figure 2C). Furthermore, some preparations crashed at very high temperatures, while others continued to oscillate. This diversity of behavior is likely the result of diverse underlying conductances in the individual pacemakers [8], [14], [21], [22]. To better understand these results, we constructed models in which we varied the maximal conductances and temperature sensitivity of the pacemaker currents, and examined how these models behaved as temperature was changed.

We chose to use a Morris-Lecar model [23] to represent the oscillator. This simple model uses biophysically realistic membrane currents and produces an oscillation that varies in amplitude, burst duration and frequency as its parameters are varied [24], and has been widely used in other studies of STG neurons and other neuronal oscillators [24]–[27]. A two-neuron multicompartmental model of the PD-AB neuron exists [28], but this model, while producing voltage trajectories that resemble those of the PD and AB neurons much more accurately than does the Morris-Lecar model, has so many parameters that it can only be studied numerically. Our intent in this paper is to understand how temperature can influence a neuronal oscillator in an analytically tractable model, and then to use these insights to guide further biological and computational studies. A single Morris-Lecar neuron [23], [24], [27] captures the features of the slow-wave oscillations that we studied experimentally, including amplitude, frequency, and duty cycle, but does not capture the fast action potentials of the biological neurons. As in previous studies [25], [27], fast action potential dynamics were neglected for two reasons. First, STG pacemaker neurons can continue to oscillate after sodium spikes are blocked (at least in some modulatory conditions) [21], [29], [30]. Second, in the STG, synaptic transmission is a graded function of presynaptic voltage [31], [32]. It therefore seems reasonable for a simple model to only capture slow-wave behavior.

The Morris-Lecar model [23] is a single isopotential electrical compartment with an instantaneous inward Ca^+2^ current, a slow outward K^+^ current, and a leak current. Here we use the same model equations to capture the total inward, outward and leak currents, respectively. While it is possible to make use of an even simpler oscillator model, we feel it is important to retain the form of actual voltage-dependent membrane conductances. The model has the following form:
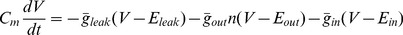
(1)

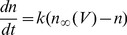
(2)where
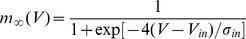
(3)

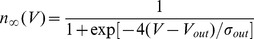
(4)
*V* corresponds to the membrane potential, *n* is the gating variable for the outward current, *k* is the maximum activation rate of *n*, *E_i_* is the reversal potential of conductance *i*, 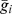 is the maximal conductance of conductance *i*, *V_i_* is the half-activation voltage for conductance *i*, *σ_i_* controls the slope of the activation curve for conductance *i*, and *C_m_* is the membrane capacitance. The capacitance, reversal potentials, steady-state activation functions and maximal conductances were chosen to roughly match known biological values [33]–[35]. The parameters were then hand-tuned to produce frequency and amplitude that resembles the pyloric output at 11°C, and were fixed unless otherwise stated (see Methods).

The electrical properties most strongly affected by temperature are the maximal conductances and the rates of channel opening and closing [5]. We added these effects to the model by making 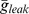, 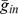, 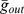, and *k* all functions of temperature, using the usual *Q_10_*-based formalism:
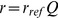
(5)

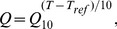
(6)where *r* is one of 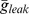, 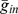, 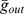, and *k*; *r_ref_* is the value of *r* at the reference temperature, *T_ref_*, here chosen to be 11°C; *Q* is the factor by which the parameter is scaled at the current temperature, *T*; and *Q_10_* describes the temperature sensitivity. Generally, the *Q_10_* values for 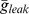, 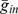, 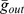, and *k* can all be different, and are denoted by 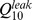, 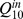, 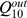 and 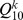, respectively. With this model we hope to illuminate temperature effects and how they relate to average inward and outward conductance dynamics, and ignore more complex contributions from large numbers of parameters in models with many conductances.

Given this model, we examined how its behavior changed as a function of temperature for different choices of *Q_10_* values and other parameters. We were particularly interested to determine whether the model was oscillatory or quiescent (non-oscillatory), and if oscillatory, what were the frequency, amplitude, and duty cycle of its membrane potential oscillations, since these are the most salient features of the biological oscillations. In the model, amplitude was defined as the peak-to-peak amplitude of the voltage waveform. Because the model did not include spikes, duty cycle was defined, somewhat arbitrarily, as the fraction of time in each cycle that the membrane potential was above the half-activation voltage of the inward conductance (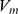), which did not vary with temperature.

In what follows, we begin with a highly constrained model and successively relax it to make it agree better with the biology. While the first model is not very realistic, it is more analytically tractable, and it yields insights that will be useful when thinking about the less-constrained models.

### Model 1: Uniform Q_10_'s yield perfect duty cycle invariance, but no crashes

**Figure 4:**
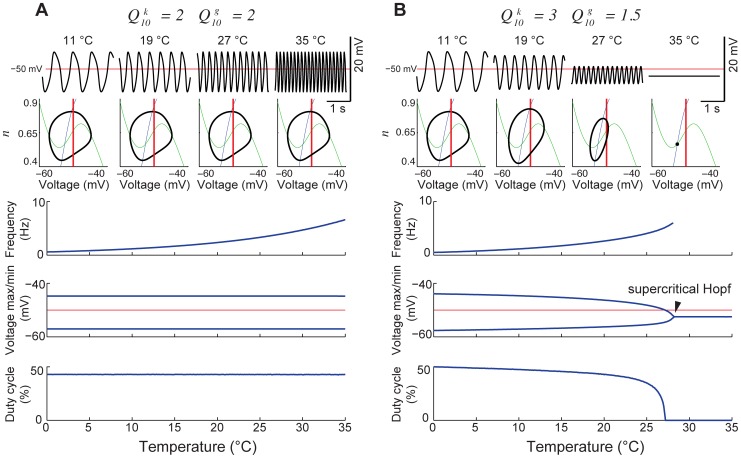
Difference in model channel temperature dependencies produces bifurcation at high temperatures. Waveforms and corresponding phase plots are plotted as examples for two different *Q_10_* relationships: (A) 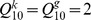 and (B) 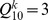, 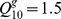. Phase plots have the gating variable *n* on the y-axis and the voltage on the x-axis. Thin green line is the V nullcline (the line where 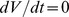) and the thin blue line is the *n* nullcline (where 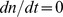). All red lines correspond to the duty cycle threshold line, chosen as the inward half activation voltage (−50 mV). Thickest black line is the limit cycle. Black dot is a stable fixed point. Lower panel plots capture simultaneous frequency, amplitude, and duty cycle plotted from 0 to 35°C with reference temperature of 11°C. Each point is calculated from the steady state solution of the model equations.

If the same *Q_10_* is used for all the temperature-dependent parameters (i.e. 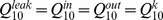), the oscillation waveform remains the same as temperature is varied, while the frequency changes according to the common *Q_10_* (Figure 4A). This implies that the duty cycle remains invariant in the face of temperature changes, which matches the pyloric pacemaker's activity (Figure 2F). However, this also implies that this model does not crash, regardless of the temperature swing, which does not match the data. We also know that in biological systems, the relevant *Q_10_*'s are not all the same [15]. Therefore we investigated models that do not require all of the *Q_10_*'s to be identical, to determine whether they would achieve approximate duty cycle invariance and also produce variable high temperature behavior.

### Model 2: Supercritical Hopf bifurcation at high temperature

Experimental measurements of channel *Q_10_* values show that channel activation rates are generally more sensitive to temperature than are maximal conductances [14]. Therefore, we next examined models in which the *Q_10_*'s for the maximal conductances remained identical (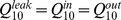), but the *Q_10_* for the activation of the outward channel, 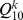, was larger. We refer to the common maximal conductance *Q_10_* as 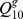. Initially, we chose 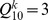 and 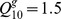, which are typical biological values [5]. With these parameters, oscillation frequency increases with increasing temperature, as before, while amplitude and duty cycle decrease slowly (Figure 4B). However, above a critical temperature the model ceases to oscillate (i.e. crashes). Note that at the temperature where oscillation ceases, the frequency is non-zero (and is, in fact, increasing up to the crash). The combination of non-zero frequency and zero amplitude transitions are hallmarks of a supercritical Hopf bifurcation. We confirmed that this transition was indeed a supercritical Hopf bifurcation for this model using numerical bifurcation analysis (see Methods). Thus, the model produced crashes similar to some of those observed biologically (Figure 3A–C), and also exhibited approximate duty cycle invariance.

**Figure 5:**
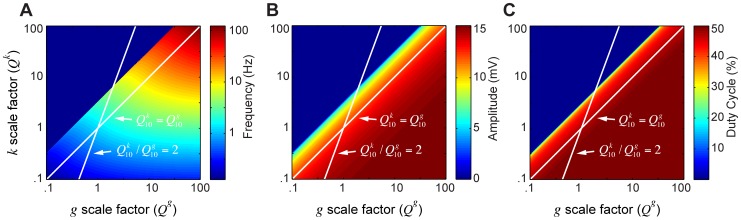
Generalized parameter scaling maps. Values from the reference model are plotted without scaling at 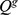 = 1 and 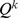 = 1. From the given reference model, along the x-axis, all conductances (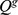) are log-scaled together; on the y-axis, the gating variable (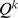) is log-scaled independently. Each point on the color plot corresponds to the measurements from the steady state model ran at their respective scaling factors. The maps of model outputs plot frequency (A), amplitude (B), and duty cycle (C). The dark blue region represents parameters where no oscillations exist. The diagonal (unity) line corresponds to a slice through parameter space where 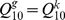, as in Figure 4A; the white line with a slope of 2 corresponds to the parameter space from Figure 4B.

To investigate whether these results might depend on our particular choice of 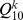 and 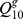, we made plots of frequency, amplitude and duty cycle as a function of two scaling factors. According to Equation 5 and 6, temperature determines a factor, *Q*. We temporarily ignore the dependence of *Q* on temperature, and treat it as an arbitrary scaling factor, which we call 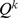 in the case of *k*, and 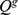 in the case of the maximal conductances. We then examine how the model behaves as a function of 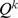 and 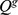, plotting both on a logarithmic scale (Figure 5). In this plot, one can see a linear boundary where frequency drops abruptly to zero (Figure 5A), and amplitude gradually decreases to zero (Figure 5B): this is a line of supercritical Hopf bifurcations. In this plot, a particular choice of 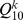 and 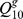 corresponds to a path through the plane, the path being parameterized by *T*. If 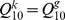 as in model 1, the line will have a slope of 1, and will never intersect the line where the bifurcation occurs, which also has a slope of 1 (this follows from the fact that when all of 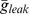, 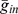, 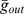, and *k* are scaled together, the model waveform does not change, only its frequency). Thus, choosing 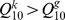 will yield a line with slope greater than 1, leading to a supercritical Hopf bifurcation when temperature is increased enough. Furthermore, and regardless of the particular choice of 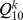 and 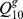, the duty cycle remains close to 50% until one is quite close to the bifurcation. Both the approximately invariant duty cycle and the crash at high temperature are general properties of model 2 as long as 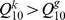.

### Model 3: Accounting for variability in pacemaker behavior at high temperatures

One unrealistic aspect of both models 1 and 2 is that frequency always increases monotonically with temperature until one reaches the crash temperature. Some experimental preparations showed a monotonic increase in frequency with temperature, but others exhibited a decrease in frequency as temperature approached the crash point (Figure 2A–C). We observed that in the model, as described by Equations 1–4 (i.e. before temperature dependence was added), increasing 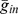 typically led to a decrease in frequency, and eventually to a crash via a fold limit cycle bifurcation (qualitatively similar to Figure 3D). Thus we reasoned that if we modified model 2 so that 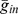 was more temperature-sensitive than 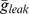 and 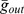, we might generate a model that, at least in some cases, had decreasing frequency as temperature was increased, and crashed via a fold limit cycle bifurcation.

**Figure 6:**
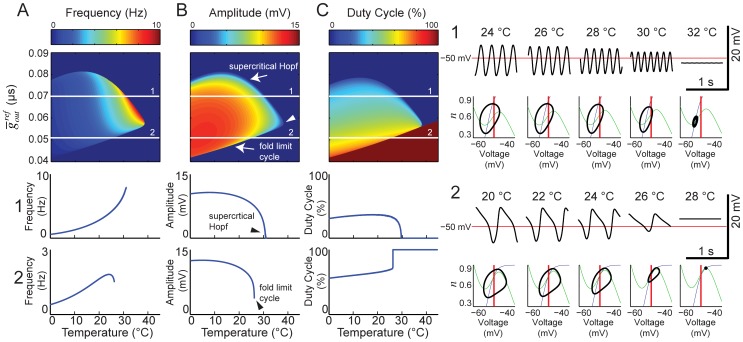
Variation in *Q_10_* ratios achieves high temperature variability in model output. Temperature dependencies in the model are as follows: 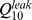 = 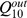 = 1.5, 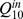 = 1.6, 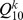 = 3. Each grid point corresponds to a model output at steady state. Temperature is plotted from 0 to 45°C, 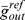 is varied from 0.04 to 0.09 µS. The maps of model outputs plot frequency (A), amplitude (B), and duty cycle (C). The amplitude map points to domains where the transition to instability happens through a supercritical Hopf or a fold limit cycle bifurcation. The mark indicates where these two bifurcations coalesce. The two white lines (1, 2) are chosen as two representative curves with qualitatively different behaviors. Line 1 is at 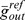 = 0.07 µS and line 2 is at 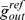 = 0.051 µS. The waveform, phase plots and scaling behavior (frequency, amplitude, duty cycle) are plotted for each line to show specific examples. For the phase plots, the thin green line is the V nullcline (where 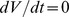) and the thin blue line is the *n* nullcline (where 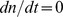). Red line is the duty cycle threshold line chosen as the inward half activation voltage (−50 mV). Black line is the limit cycle.

We therefore implemented model 3, in which 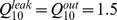, 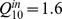, and 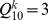. We studied the model's output over a range of maximal outward conductance at the reference temperature (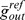), while fixing all other parameters (Figure 6). There were two qualitatively different possibilities for the oscillator behavior as temperature was varied. At high values of 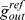, we observed a monotonically increasing frequency curve, amplitude decreasing to zero, and eventually a supercritical Hopf bifurcation (Figure 6, case 1), very similar in behavior to model 2. In this case, the limit cycle drifted below the duty cycle threshold and eventually dropped to 0% as a function of temperature. Low values of 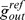, on the other hand, yielded a decrease in frequency as the crash was approached, and a fold limit cycle bifurcation (Figure 6, case 2). In this case, the limit cycle drifted upwards relative to the duty cycle threshold, causing the duty cycle to monotonically increase and eventually reach 100%.

Thus model 3 mimics some of the individual-to-individual variability seen in the biological data. Depending on the parameters, it can produce either a monotonic or an inverted U-shaped F-T curve (compare Figure 6A, bottom two panels, with Figure 2C). It can also produce two different kinds of crashes, mimicking the dichotomy seen experimentally (Figure 3). In both cases it produces approximate duty-cycle invariance, as is observed experimentally (compare Figure 6C with Figure 2F).

### Extreme temperatures can reveal underlying individual-to-individual differences

We know that despite great variability in underlying parameters, pyloric rhythm output is extremely consistent from animal-to-animal at moderate temperatures, but diverges at extreme temperatures [15]. Likewise, the frequency of the pyloric pacemakers is highly constrained at moderate temperatures, but widely variable at high temperatures (Figure 2D). Thus high temperatures can reveal differences between individuals that are obscured at normal temperatures. We were curious whether varying the parameters of model 3 could yield this pattern of behavior: low variability at moderate temperature but high variability at high temperature.

**Figure 7:**
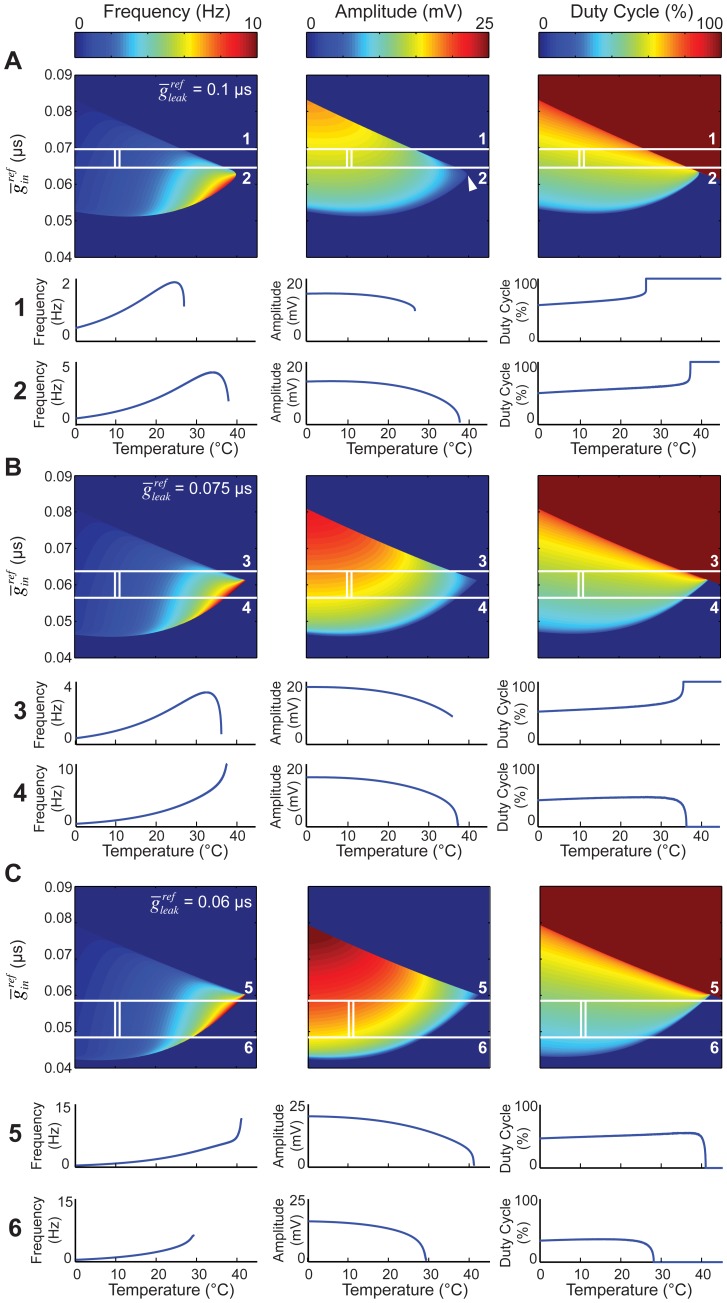
Restricting low temperature frequency output constraints high temperature variability. Three models were generated with the same reference parameters, but different 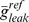 values. Temperature dependence fixed at: 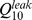 = 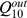 = 1.5, 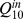 = 1.6, 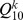 = 3. Frequency, amplitude and duty cycle maps were generated (as in Figure 5) for the three parameter points, varying temperature and inward conductance: (A) 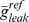 = 0.1 µS, (B) 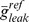 = 0.075 µS, and (C) 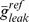 = 0.06 µS. The white boxes constrain a region from 10 to 11°C where frequency is between .95 and 1.05 Hz. The horizontal lines represent the vertical boundaries of the box and are plotted explicitly below each map to demonstrate the high temperature variability. Line 1 – 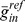 = 0.0696 µS; line 2 – 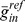 = 0.0645 µS; line 3 – 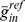 = 0.0639 µS; line 4 – 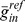 = 0.0563 µS; line 5 – 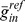 = 0.0587 µS; line 6 – 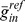 = 0.0486 µS. White mark corresponds to bifurcation coalescence point that defines the parameter region for Figure 8.

We examined three versions of model 3, with different maximal leak conductances at the reference temperature (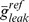; 0.1, 0.075, and 0.06 µS) but all other parameters fixed (Figure 7). For each model, we determined the range of 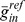 values that would yield a frequency between 0.95 and 1.05 Hz for all temperatures between 10 and 11°C (narrow vertical rectangles in the parameter maps of Figure 7). We then varied the temperature for the models corresponding to the highest and lowest values of 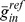 in each case (horizontal white lines in Figure 7), and examined the pacemaker behavior at high temperatures. For the highest value of 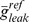, we observed inverted U-shaped F-T curves in both cases, and both models crashed via a fold limit cycle bifurcation, but they did so at different temperatures (Figure 7A). For the middle value of 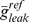, one F-T curve was monotonic, and the other was inverted U-shaped (Figure 7B). The former model crashed via a supercritical Hopf bifurcation, the latter via a fold limit cycle bifurcation, and their frequencies just below the crash point were very different. For the lowest value of 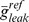, both models yielded monotonic F-T curves and supercritical Hopf bifurcations, but both their crash temperatures and their frequency just shy of the crash were quite different (Figure 7C). Thus the model demonstrates how individuals that behave similarly at moderate temperatures can diverge considerably at extreme temperatures.

**Figure 8:**
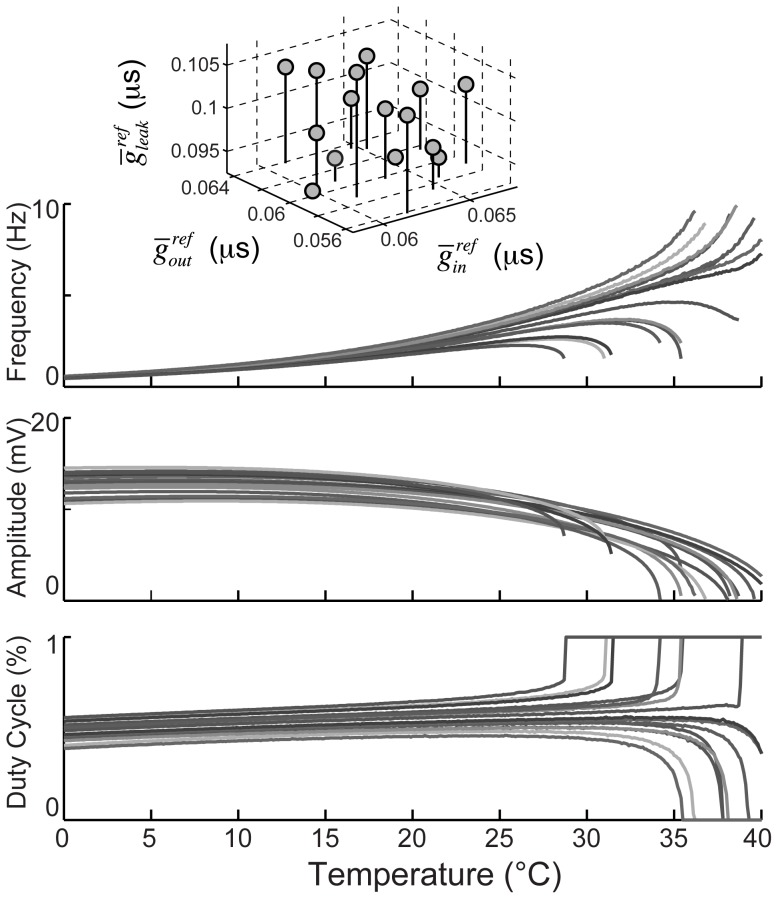
Example of random parameter choices producing similar output at low temperature and divergent output at high temperature. Temperature dependence fixed at: 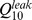 = 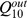 = 1.5, 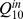 = 1.6, 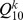 = 3. From a chosen reference parameter point, 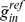 = 0.063 µS; 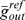 = 0.06 µS; 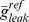 = 0.1 µS, at 11°C, each maximal conductance (leak, inward and outward) is given a ±7.5% tolerance and 15 randomly generated curves are plotted across temperature. Variability in parameter space is shown in the 3D plot above. The frequency, amplitude and duty cycle of the 15 models are plotted simultaneously across the three graphs as a function of temperature.

To further demonstrate the variability that can arise from small differences in underlying reference-temperature parameters, we picked random values for 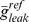, 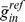, and 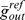 from a range that covered the intersection of the two bifurcations shown in Figure 7A (white arrow). This sample of models yielded similar frequency, amplitude and duty cycle near the reference temperature (11°C), but their behavior diverged as temperature was increased (Figure 8). This phenomenon is similar to that observed for the biological pacemaker frequency (Figure 2C). Again, this demonstrates how individuals that behave similarly at moderate temperatures can diverge wildly at extreme temperatures.

## Discussion

Neuronal oscillators are ubiquitous throughout the nervous system. Some individual neurons are intrinsically oscillatory, by virtue of their voltage-dependent conductances [22], [36]–[38]. Other oscillations arise from network interactions, such as reciprocal inhibition [39]–[42] or other circuit configurations [43]. Rhythmic pattern generators must maintain robust and reliable activity in the face of external and internal perturbations. For the central pattern generators that control movement, it is important to control both the frequency of the motor pattern as well as the phase relationships of all of the constituent circuit elements [44]. Mathematical models have been quite instructive for understanding many features of central pattern generating circuits. For example, in many circuits reciprocal inhibition helps ensure the out-of-phase activity of functional antagonists, but it was theoretical models that illuminated the richness of behaviors possible with reciprocally inhibitory circuits [42], [45]. In one of the best-understood circuits driven by reciprocal inhibition, the leech heartbeat system, mathematical models and bifurcation analysis have been particularly helpful in understanding the parameters that allow the system to be sensitive to various modulator inputs while maintaining robust activity [46]–[48].

In general, studying the stability of oscillators by perturbing them in a realistic fashion, especially to the point of failure, can help reveal the salient features that contribute to their robustness. In this paper, we use temperature to perturb the pacemaker of the pyloric circuit of the stomatogastric ganglion, to assess the extent to which the mechanisms that give rise to this function are variable across individual animals. The responses of oscillators to perturbations have been extensively studied and characterized using dynamical systems theory [20], [47], [49]–[53]. As a result, it was possible to use a mathematical framework to loosely infer the underlying structure of the pyloric pacemaker responsible for generating the behavior produced in response to temperature. This kind of analysis is particularly revealing at points of instability or bifurcation as it offers qualitative points of comparison between the biological oscillator and the model. The temperature-induced bifurcations in the models studied here were of two forms, supercritical Hopf and fold limit cycle. In the supercritical Hopf bifurcation, the oscillation amplitude decreases gradually, but the oscillation stops at a non-zero frequency (Figure 6A). In the fold limit cycle bifurcation, the oscillation does not gradually decrease in amplitude, but stops abruptly from a non-zero amplitude (Figure 6B). The bifurcations in the model can be unambiguously identified because all the state variables are known, as are their dynamics. Although the biological pacemakers show behaviors that look similar to those of the models, we presently lack detailed knowledge of the full complement of voltage- and time-dependent currents in the biological PD and AB neurons. A complete understanding of how temperature affects the biological oscillator must await biophysical studies of the effects of temperature on each of the currents in these neurons, followed by the construction of a far more biophysically realistic computational model that incorporates those data.

Nonetheless, we can account for the temperature compensation of phase in the intact pyloric rhythm as a consequence of two processes: the maintenance of pacemaker duty cycle shown here and the effect of temperature on the follower neurons [14]. Interestingly, the pacemaker duty cycle's relative invariance to period depends on the interaction between the AB and PD neurons [25], [28], so temperature must produce balanced effects on the AB and PD neurons and the electrical coupling between them.

The pacemaker neurons provide strong inhibitory drive to the follower neurons, which imposes a well-defined temporal constraint on them as temperature is changed [14]. Because the LP and PY neurons burst on rebound from inhibition [54]–[57], the phase invariance of the LP and PY neuron's activity is partially accounted for by the phase invariance of the pacemaker [14], [15]. Additionally the intrinsic I_A_ and I_h_ conductances in the LP neuron scale with temperature in a complementary way that also contributes to the temperature invariance of the pyloric phase relationships [14]. The combination of the pacemaker's intrinsic stability and the synaptic and intrinsic channel regulation of the follower neurons explains how phase-temperature stability is achieved at the level of the entire network.

Pacemaker duty cycle stability arises from the scaling of the limit cycle appropriately around the duty cycle threshold. We can speculate that in the biological system, the limit cycle as well as the duty cycle threshold change in such a manner as to conserve these relationships in response to temperature perturbation. Furthermore, the model results inform the understanding of how duty cycle reliability is consistent with diversity in behavior at high temperatures (Figures 6–8). While the intrinsic temperature-dependent model parameters are well balanced at low temperatures, they diverge steeply at higher temperatures because of the variable exponential temperature-dependent terms. Of course, the Morris-Lecar model is, at best, an over-simplified caricature of the biological pacemaker. Furthermore, in the work presented here, temperature dependence was implemented as a simplified approximation of the full range of temperature's effects on ion channel function. Nevertheless, the model's simplicity allows for intuitive, testable predictions regarding channel temperature dependencies and deepens our understanding of the way in which variability plays a role in robustness.

Not all biological circuits routinely face large swings in temperature. Yet, all networks are challenged with perturbations of their internal or external environment. Because there are many sets of underlying parameters that can give rise to similar neuronal or network performance [9], [58]–[63], it is critical for us to understand how network robustness is maintained in the face of perturbations across a population of individuals with variable sets of network parameters. The work presented here provides one example of the remarkable robustness that biological networks can display over a large range of parameters. Nonetheless, it is important that the parameters underlying robust network performance in a population of wild-caught crabs are the result of long years of evolutionary pressure, and we understand as yet little of the mechanisms by which individual animals find one of the sets of parameters that can allow it to live for years in a variable world.

## Methods

### Animals

*Cancer borealis* were purchased from Commercial Lobster (Boston, MA). The tanks were kept at approximately 7°C, 11°C, or 19°C. The experiments reported in this manuscript were done between December 2010 and April 2011.

### Solutions

*C. borealis* physiological saline was composed of 440 mM NaCl, 11 mM KCl, 13 mM CaCl_2_, 26 mM MgCl_2_, 11 mM Trizma base, and 5 mM Maleic acid, pH 7.4–7.5. The microelectrode solution contained 0.6 M K_2_SO_4_ and 20 mM KCl. Picrotoxin (PTX) was obtained from Sigma and used at 10^−5^ M in saline.

### Electrophysiology

The stomatogastric nervous system was dissected out of the animals and pinned out in a Sylgard (Dow Corning) coated plastic Petri dish containing chilled saline at 11–12°C [64]. During experiments, the preparations were continuously superfused with saline. Isolated pacemaker experiments were done in 10^−5^ M PTX, which blocks glutamatergic synapses in crustaceans [17], [18]. The PD, LP and PY neurons project axons to the *pdn*, *gpn* and *pyn*, respectively. Vaseline wells were placed around the corresponding motor nerves and extracellular recordings were obtained using stainless steel pin electrodes placed in the wells and amplified using a differential amplifier (A-M Systems). Intracellular recordings were obtained from STG somata using 10–30 MΩ glass microelectrodes pulled with a Flaming/Brown micropipette puller (Sutter Instrument Company). For all intracellular recordings, the STG was desheathed. Neurons were identified using previously described procedures [65].

The temperature of the saline was controlled using a Peltier device (Warner Instruments), which had a precision of ±0.5°C. Slight adjustments of the electrode position were required with large swings of temperature, as the cells tended to swell at high temperatures. Temperature was increased from 11 to 31°C in increments of 4°C. Each preparation was given at least 5 minutes to adapt at a new steady-state temperature before taking data. At the end of each experiment, the temperature was returned to the reference 11°C to check that no irreversible changes had occurred and that the circuit output remained the same.

### Data analysis

Data were acquired using a Digidata 1200 data acquisition board (Axon Instruments) and analyzed using Clampfit 9.0 (Axon Instruments), Spike 2.5 (Cambridge Electronic Design), and MATLAB 7.1 (Mathworks). All figures were generated in Adobe Illustrator CS5, and obvious electrical artifacts were removed by hand. Spike2 scripts written by Dirk Bucher were used to extract phase and frequency from extracellular recordings [65].

To quantify the temperature sensitivity of a quantity (e.g. pyloric frequency), we calculated the *Q_10_*. That is, we assumed the quantity, *r*, fit the equation
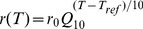
(7)as a function of temperature, where all parameters are as in Equations 5 and 6. We then fit a line to a plot of 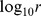 versus temperature, *T*, and extracted the slope of this line, *m*. The *Q_10_* is then given by
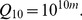
(8)


The PD neuron's duty cycle was calculated as the ratio of burst duration to period. Burst period was the time between PD burst onsets, and frequency as the reciprocal of the period. Data from 8 experiments with and 6 experiments without intracellular recordings were pooled for analyzing average frequency and duty cycle. A rhythm was considered crashed when periodic oscillatory bursting behavior terminated. All statistical analyses were performed using SigmaPlot and SigmaStat 11.0 software packages (Jandel Scientific). XPPAUT 5.6 was used to analyze and characterize bifurcations in the Morris-Lecar model for all the parameter sweeps preformed in the paper [49]. Matlab was used to numerically calculate the model output and generate the parameter color maps.

For performing statistical comparisons of relative variability, we used the sample variance of the log-transformed data (denoted S^2^_log_ in Figure 2D) instead of the coefficient of variation. This provides a measure of variability which is invariant to the overall scale of the data (like the coefficient of variation), but because it is a sample variance it has more convenient statistical properties [66].

### Model

The Morris-Lecar model is defined by Equations 1–4. The model used in this work has some differences from the original Morris-Lecar model [23]: the *I_app_* term is dropped, and in our hands the two currents are intended to describe an aggregate inward and an aggregate outward current, rather than being literal calcium and potassium currents. All reference model parameters are fixed unless otherwise stated; parameters were chosen to resemble real biological values: 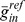 = 0.06 µS; 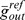 = 0.06 µS; 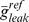 = 0.1 µS; *E_in_* = −10 mV; *E_out_* = −80 mV; *E_leak_* = −50 mV; *k* = 3 Hz; *σ_in_* = 10; *σ_out_* = 7; *V_in_* = −50 mV; *V_out_* = −53 mV; *C_m_* = 5 nF.
